# Socioeconomic and environmental determinants of foot and mouth disease incidence: an ecological, cross-sectional study across Iran using spatial modeling

**DOI:** 10.1038/s41598-023-40865-4

**Published:** 2023-08-19

**Authors:** Mahdi Nazari Ashani, Ali Asghar Alesheikh, Zeinab Neisani Samani, Aynaz Lotfata, Sayeh Bayat, Siamak Alipour, Benyamin Hoseini

**Affiliations:** 1 Department of Geospatial Information Systems, Faculty of Geodesy and Geomatics Engineering, K. N. Toosi University of Technology, Tehran, Iran; 2 Department of Pathology, Microbiology, and Immunology, School of Veterinary Medicine, University of California, Davis, USA; 3 Department of Biomedical Engineering, University of Calgary, Calgary, AB Canada; 4 Department of Geomatics Engineering, University of Calgary, Calgary, AB Canada; 5 Pharmaceutical Research Center, Pharmaceutical Technology Institute, Mashhad University of Medical Sciences, Mashhad, Iran; 6 Department of Medical Informatics, Faculty of Medicine, Mashhad University of Medical Sciences, Mashhad, Iran

**Keywords:** Climate sciences, Ecology, Risk factors

## Abstract

Foot-and-mouth disease (FMD) is a highly contagious animal disease caused by a ribonucleic acid (RNA) virus, with significant economic costs and uneven distribution across Asia, Africa, and South America. While spatial analysis and modeling of FMD are still in their early stages, this research aimed to identify socio-environmental determinants of FMD incidence in Iran at the provincial level by studying 135 outbreaks reported between March 21, 2017, and March 21, 2018. We obtained 46 potential socio-environmental determinants and selected four variables, including percentage of population, precipitation in January, percentage of sheep, and percentage of goats, to be used in spatial regression models to estimate variation in spatial heterogeneity. In our analysis, we employed global models, namely ordinary least squares (OLS), spatial error model (SEM), and spatial lag model (SLM), as well as local models, including geographically weighted regression (GWR) and multiscale geographically weighted regression (MGWR). The MGWR model yielded the highest adjusted ${R}^{2}$ of 90%, outperforming the other local and global models. Using local models to map the effects of environmental determinants (such as the percentage of sheep and precipitation) on the spatial variability of FMD incidence provides decision-makers with helpful information for targeted interventions. Our findings advocate for multiscale and multidisciplinary policies to reduce FMD incidence.

## Introduction

Foot and mouth disease (FMD) has been recognized as a significant epidemic with severe implications for the livestock industry since the sixteenth century^1^. FMD virus spreads through direct or indirect contact with infected animals' secretions or excretions, inhalation, ingestion, skin wounds, and mucous membranes. It causes blisters in and around the mouth, feet, and udders of animals, as well as fever, lameness, loss of condition, milk drop in dairy cattle, and sudden death in young stock due to myocarditis^2^. The FMD virus poses a substantial risk, often leading to widespread outbreaks because of its highly contagious nature. The 2001 FMD outbreak in England is an example, where approximately 6.7 million animals were slaughtered in the United Kingdom and the Netherlands, resulting in significant economic losses amounting to around 3.2 billion euros^3,4^.

While FMD has been eradicated in some parts of the world, including North America and Europe, it remains endemic in some areas in Asia, Africa, and the Middle East. Most of the economic burden of this infectious epidemic in these countries was borne by lost trade and large numbers of infected animals^2^. The global distribution of FMD virus serotypes is categorized into seven pools. Pools 1 and 2 encompass Southeast and Southern Asia, respectively, while Pool 3 includes Euro-Asia (including the Middle East). Pools 1, 2, and 3 collectively harbor three circulating FMD virus serotypes O, A, and Asia 1. The remaining four pools, namely Pool 4, Pool 5, Pool 6, and Pool 7, represent other regions worldwide where the FMD virus is prevalent^5^.

Recent studies^6–8^ have revealed that various factors may contribute to the severity of FMD. Arman et al.^6^ identified a higher number of FMD epidemics during the driest season in Ethiopia, suggesting that seasonal changes may be a risk factor for FMD in the region. Jiang et al.^7^ studied the climate change impacts on the FMD risk in elephants using Maximum Entropy (MaxEnt). Their study revealed that temperature seasonality, average annual temperature, average temperature in the driest quarter, and precipitation in the driest month were the most significant factors influencing FMD risk. A study conducted in Ethiopia by Udahemuka et al.^8^ showed that vaccinating calves younger than 12 months effectively control FMD within the herd. These findings contribute to our understanding of the risk factors associated with FMD and offer valuable insights for developing effective control and prevention measures.

Spatial analysis is a valuable tool for studying the distribution of infectious diseases^9–11^. Ballard and Boone^12^ used geographically weighted regression (GWR) to examine the relationship between Lyme disease and land cover in the Midwestern and Northeastern states of the United States. Some studies have used spatial analysis to model FMD^13–17^. Sangrat et al.^13^ utilized a multi-criteria decision analysis method based on geographic information system (GIS) to predict FMD-prone areas in Thailand, identifying regions such as the northern, northeastern, western, and central parts of the country as FMD hotspots. Gao et al.^14^ investigated the spatial distribution and risk zones of FMD in mainland China, identifying significant factors such as road density, isothermality, UV-B radiation seasonality, minimum temperature in the coldest month, market distribution, and railway density. Hagerman et al.^15^ studied seasonal and geographic conditions favorable to the airborne spread of FMD in the United States, highlighting high-risk geographical areas, particularly during seasons with favorable weather conditions. These studies demonstrate the significance of spatial analysis in understanding and managing the spatial patterns and risks associated with infectious diseases.

Spatial analysis has been employed in a few studies to model FMD in Iran^16,17^. Ilbeigi et al.^18^ identified Razavi Khorasan province as having a high risk of FMD prevalence due to a lack of regular on-farm hygiene control. They found that the lack of biosecurity was 11 times higher in FMD-infected farms compared to non-infected farms^18^. Jafarzadeh et al.^16^ used a zero-inflated negative binomial model to estimate the probability of a "no outbreak" state and the number of outbreaks in Iran at the province level, utilizing data from 5707 FMD cases recorded between April 1995 and March 2002. Similarly, Perez et al.^17^ employed spatial scan statistics to examine the spatial distribution of FMD in Iran using 4477 FMD cases reported from June 1996 to September 2003. Their analysis identified significant clusters of FMD that coincided with road networks, neighboring countries, and densely populated areas^17^.

This study investigates the determinants and their significance in influencing provincial-level FMD incidence across Iran. The study's objectives encompass several aspects, including conducting exploratory analysis to identify the determinants of FMD incidence, examining the geographical variations of these determinants using local spatial modeling techniques, and analyzing FMD spatial variations through both aspatial and spatial regression models. Notably, this research represents the first study to compare local modeling with global modeling approaches in assessing the distribution of FMD across Iran. By addressing these objectives, this study provides valuable insights into the factors driving FMD incidence at the provincial level. It contributes to a better understanding of the spatial patterns of FMD in Iran.

## Methods

### Study area

**Figure 1:**
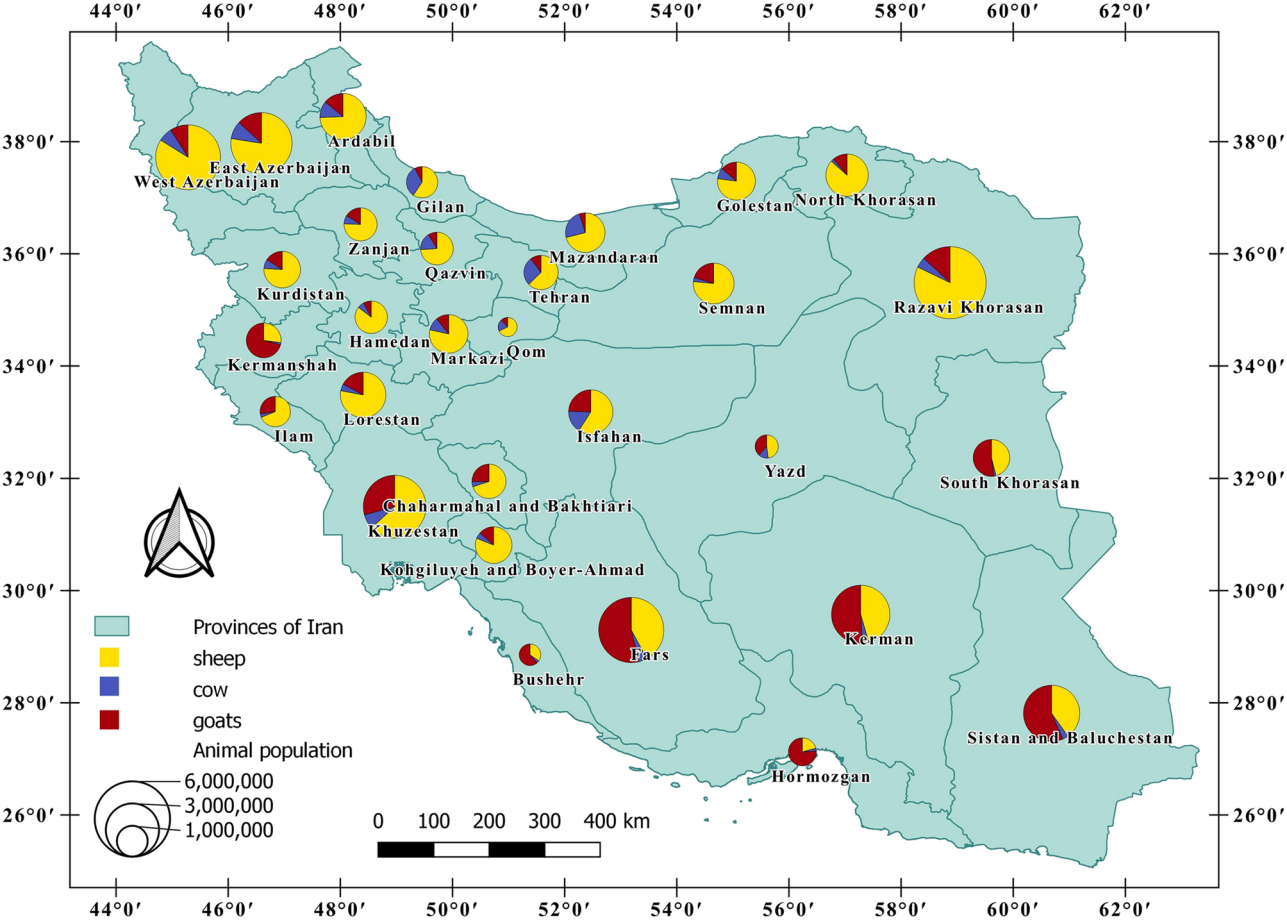
The animal population per province in Iran.

Iran, located in the Middle East (32.4279° N, 53.6880° E), is the focal point of this study as a representative case study within Asia. The country is divided into 31 provinces, each characterized by varying precipitation levels, temperature ranges, and diverse climates, including hot, dry, moderate, and humid conditions. Iran boasts a diverse topography, ranging from regions below sea level to mountainous areas reaching heights of approximately 5600 m above sea level^19^. The average province size is around 20,871.9197 square miles, with an average population of 2,573,796. Provinces were selected as the analytical units for this study, considering data availability and relevance. Figure 1 visually depicts the animal population distribution across Iran's provinces, providing valuable insights into the spatial patterns of animal population density.

### FMD incidence as the outcome variable

**Figure 2:**
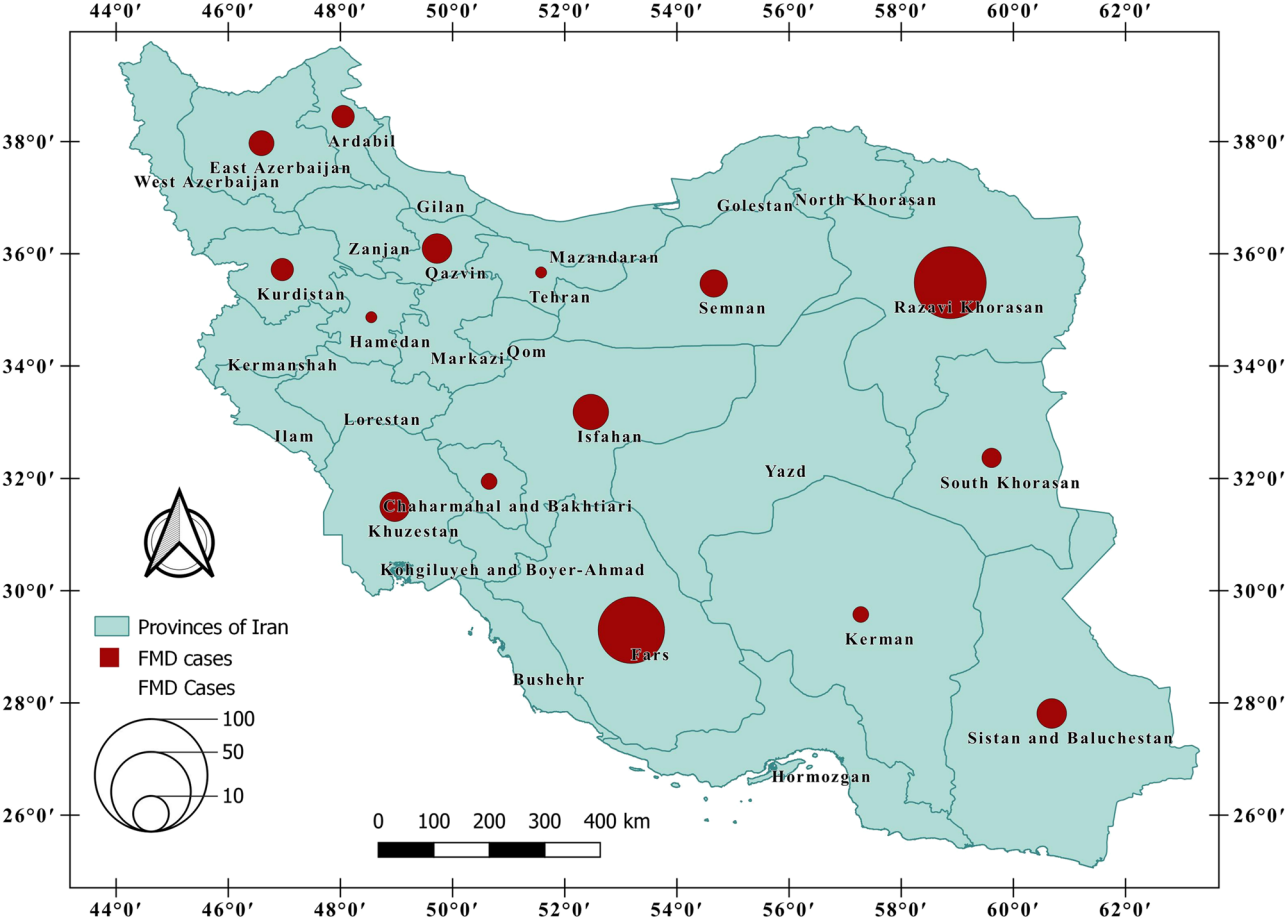
Illustrating the FMD cases from March 21, 2017 to March 21, 2018.

Animal FMD cases for 30 census provinces in Iran (see Fig. 2) were obtained from the Iran Veterinary Organization (IVO). The province of Alborz was excluded from the analysis due to insufficient data availability. For this study, the outcome variable was the incidence of FMD calculated as the number of cases per 1,000,000 animals in Iran's provinces between March 21, 2017, and March 21, 2018.

### Covariates

In this study, we evaluated 46 potential socio-environmental determinants (see Table 1). Among these determinants, we selected four variables, namely the percentage of population, precipitation in January, percentage of sheep, and percentage of goats, to be used as covariates in the spatial regression models. The data for these variables were sourced from multiple reliable sources to ensure comprehensive coverage and maintain the accuracy of our analyses. In the first step, we obtained data on the percentage of the population, educated individuals, and migrants from the Population and Housing Census of Iran^20^. The unemployment rate per province was acquired from The Summary of the Results of the 2016 Workforce Statistics Plan of Iran^21^. In the second step, we collected data on the percentage of sheep, goats, and cows per province from the National Agricultural Census of Iran^22^. Third, we gathered data on the percentage of the Gross Domestic Product (GDP) per province from the Iran Centre of Statistics Ministry of Cooperatives, Labor, and Social Welfare for the period between March 21, 2017, and March 21, 2018^23^. Fourth, we obtained data on the monthly minimum and maximum temperatures for the provinces from March 2017 to March 2018, provided by the Iranian Metrological Organization^24^. This data was initially in tabular format for a list of weather stations across Iran. To obtain temperature values at the provincial level, we employed the inverse distance weighted (IDW) method^25^. We generated interpolated temperature rasters covering the entire country using IDW. Subsequently, we calculated the average of the minimum and maximum temperatures for each month per province, utilizing the results obtained from the IDW interpolation process in the previous step. Fifth, we acquired monthly precipitation data^26^ from the Climate Research Unit-Time Series, downscaled to a spatial resolution of 2.5 min (~ 21 km^2^) using WorldClim 2.1^27^. This dataset consists of 12 raster files spanning from March 2017 to March 2018, with precipitation values provided in millimeters. To derive the monthly average precipitation at the provincial level, we aggregated the raster data from March 2017 to March 2018 for each province.

**Table 1:** Definitions and sources of explanatory variables used in this study.

Theme	Variable	Description	Source
Socioeconomic	1. Unemployment rate 2. Education 3. Migration 4. GDP (without oil)	1. Unemployment rate: $\frac{{\text{Number}} \, {\text{of}} \, {\text{unemployed}} \, {\text{in}} \, {\text{province}}}{{\text{Total}} \, {\text{population}} \, {\text{in}} \, {\text{province}}}\times 100$ 2. Percentage of educated persons: $\frac{{\text{Number}} \, {\text{of}}\text{ educated }{\text{in}} \, {\text{province}}}{{\text{Total}} \, {\text{population}} \, {\text{in}} \, {\text{province}}}\times 100$ 3. Percentage of migrated persons: $\frac{{\text{Number}} \, {\text{of}}\text{ migrants }{\text{in}} \, {\text{province}}}{{\text{Total}} \, {\text{population}} \, {\text{in}} \, {\text{province}}}\times 100$ 4. Percentage of gross domestic product (GDP) without oil from March 21, 2017, to March 21, 2018	1. The summary of the results of the 2016 workforce statistics plan of Iran^21^ 2, 3. Population and housing census of Iran, 2016^20^ 4. An overview of the gross domestic product by province from 2010 to 2018^23^
Demographic	1. Population 1. Sheep 2. Goats 3. Cows	1. Percentage of population: $\frac{{\text{Number}} \, {\text{of}}\text{ population }{\text{in}} \, {\text{province}}}{{\text{Total}} \, {\text{population}} \, {\text{in}}\text{ the country}}\times 100$ 1. Percentage of sheep: $\frac{{\text{Number}} \, {\text{of}}\text{ sheep }{\text{in}} \, {\text{province}}}{{\text{Total}}\text{ sheep }{\text{in}}\text{ the country }}\times 100$ 2. Percentage of goats: $\frac{{\text{Number}} \, {\text{of}}\text{ goats }{\text{in}} \, {\text{province}}}{{\text{Total}}\text{ goats }{\text{in}}\text{ the country}}\times 100$ 3. The percentage of cows: $\frac{{\text{Number}} \, {\text{of}}\text{ cows }{\text{in}} \, {\text{province}}}{{\text{Total}}\text{ cows }{\text{in}}\text{ the country}}\times 100$	1. Population and Housing Census of Iran, 2016^20^ 2, 3, 4. National Agricultural Census of Iran, 2014^22^
Environmental	1. Temperature 1. Precipitation 2. NDVI	1. Minimum and maximum for each month (°C) (24 variables) 1. Average precipitation for each month (mm) (12 variables) 2. Normalized difference vegetation index (NDVI)	1. Monthly synoptic data of Iran from March 2017 to March 2018^24^ 1. CRU-TS 4.03^26^ downscaled with WorldClim 2.1^27^ 1. Obtained from NASA Earth Observation, (2022)^29^
Topographic	Elevation	DEM of the U.S. (30 m spatial resolution)	Obtained from National Geospatial-Intelligence Agency, (2022)^28^

Sixth, The National Geospatial-Intelligence Agency^28^ supplied the Digital Elevation Model (DEM) with a resolution of 30 m. DEM was employed to calculate the average elevation per province. Lastly, NASA Earth Observations^29^ provided the monthly Normalized Difference Vegetation Index (NDVI). The average NDVI value per province was derived for each 12 months from March 2017 to March 2018. The NDVI values in this study ranged from − 1 to 1, where a value of 1 indicated higher vegetation. They were rescaled to a range of 0–255 and stored as unsigned 8-bit data for data storage.

### Descriptive and exploratory analysis

First, the Pearson correlation matrix was applied to explore the correlation between all 46 explanatory variables and remove explanatory variables with low correlation to FMD incidence (dependent variables). Explanatory variables with correlation coefficients less than |0.3| (poor correlation^30^) were removed from the modeling process (see Supplemental Fig. 1). Then, the variance inflation factor (VIF) was employed to detect variables with a high VIF, more than 5, to remove exploratory variables with high multicollinearity^31^. For comparison, all global and local methods were implemented with the same chosen variables. We executed a global Moran I statistic to find the pattern of FMD incidence using ArcMap (version 10.8).

**Figure 3:**
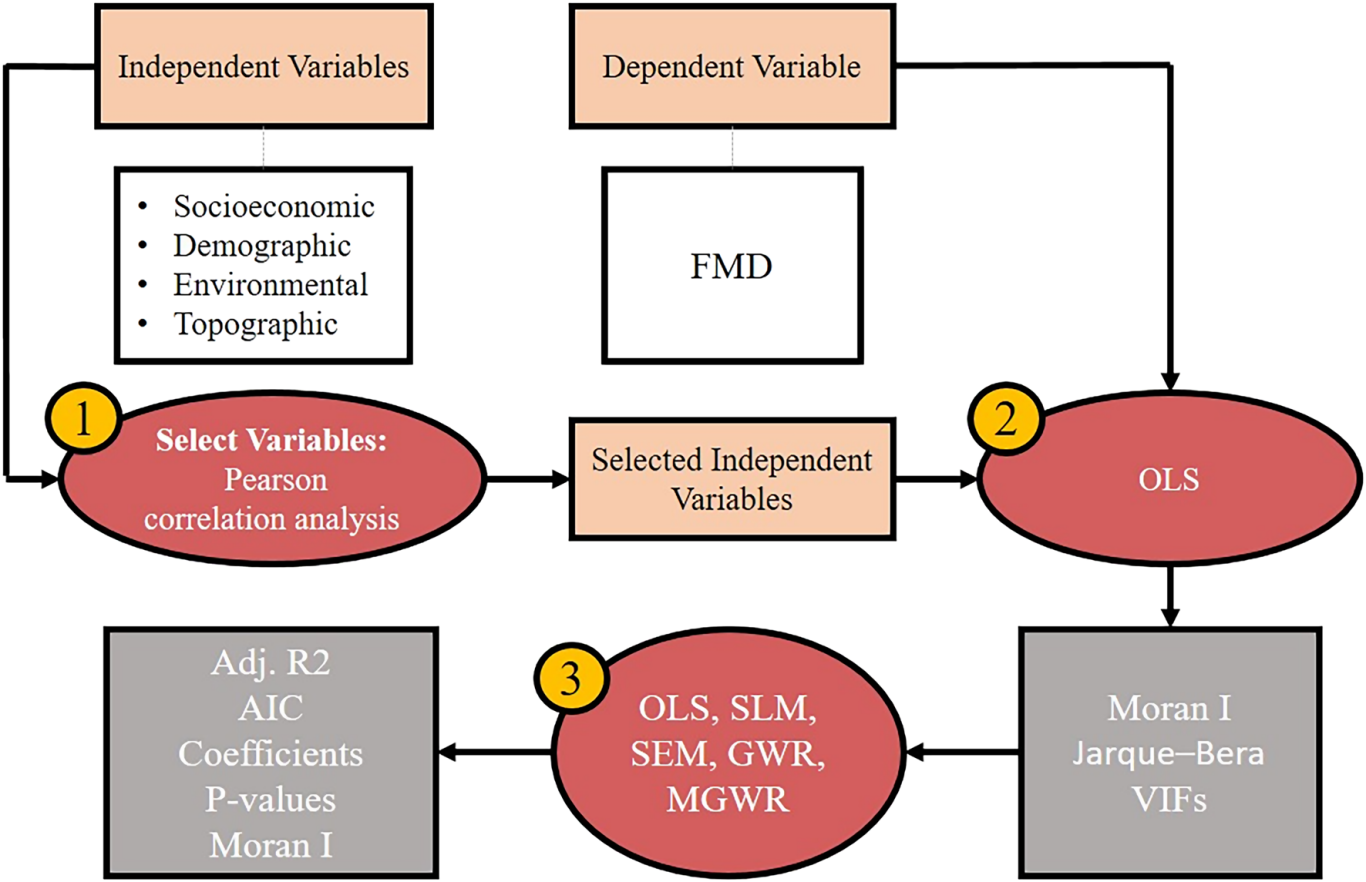
Illustrating workflow of the study.

Next, Global models were executed by Python Spatial Library (PySal) in a Python environment (version 3.7.11) using the first-order Queen contiguity to calculate the weight matrix^32^. Then, Local models were executed by MGWR (version 2.2) using a Fixed Gaussian kernel, set with the bandwidth and minimized corrected Akaike's Information Criterion (AIC)^33^. Finally, adjusted ${R}^{2}$ and AIC were used to examine the performance of the models, and the Moran I test for GWR and MGWR residuals were computed to test the models’ residual spatial autocorrelation. Figure 3 indicates the workflow of the study.

### Global models

Three global regression models were applied in this study, including ordinary least square (OLS), spatial lagged model (SLM), and spatial error model (SEM). OLS is a linear aspatial regression method that can estimate the dependent variable (incidence of FMD) using a group of independent variables^34^. SLM is a subset of the OLS^35^ that considers the effect of a spatial unit on adjacent units in the region, as shown in the Eq. (1)^36,37^. SLM explains as follows:

1 $${y}_{i}={b}_{0}+{x}_{i}b+\rho {w}_{i}{y}_{i}+{e}_{i}$$

*ρ* is the spatial auto-regressive ratio, and w is the spatial weight matrix representing distance associations between the centroid of provinces. The spatial lag function that assesses the effect of adjacent variables together can involve an independent variable during modeling^36^. In the SEM model, ${e}_{i}$ is the absolute error term responsible for solving the problem of spatial autocorrelation. It splits into two components: first, the spatial component of the error term ($\lambda {w}_{i}{\xi }_{i}$), and second, random error (${e}_{i}$) as follows^38^:

2 $${y}_{i}={b}_{0}+{x}_{i}b+\lambda {w}_{i}{\xi }_{i}+{e}_{i}$$

### Local models

Geographically weighted regression (GWR) and multiscale geographically weighted regression (MGWR) are two local spatial regression models used in this study. GWR donates to modeling spatial processes, and it can estimate the dependent variable (FMD incidence) using a group of independent variables measured at a location^39^. The equation of GWR is as follows:

3 $${y}_{i}\left(u\right)={\beta }_{0i}\left(u\right)+{\beta }_{1i}\left(u\right){x}_{1i}+{\beta }_{2i}\left(u\right){x}_{2i}+\cdots +{\beta }_{mi}\left(u\right){x}_{mi}$$

${y}_{i}\left(u\right)$ is the dependent variable, in this case study, FMD incidence. The parameters with the notation ${x}_{ji}$ are independent variables, for example, the number of sheep or precipitation in location i. In Eq. (3), we see the number of independent variables is m. ${\beta }_{0i}$ shows the parameter that explains a relationship around location u and is specific to that location $\beta ^{ \wedge } (u)$ takes the form as follows:

4 $$ \beta ^{ \wedge } (u) ={\left({X}^{T}W\left(u\right)X\right)}^{\left(-1\right)}{X}^{T}W\left(u\right)y$$

*W* represents the weight matrix that considers the effect of the neighboring points relative to point u. ${X}^{T}W\left(u\right)X$ is the geographically weighted variance–covariance matrix, and y is the vector of the values of the dependent variable (FMD incidence in this research). The leading diagonal of the $W\left(u\right)$ matrix consists of the geographical weights, and the off-diagonal elements of the $W\left(u\right)$ matrix are 0. The weights computed using the Gaussian kernel^39^ as follows:

5 $${w}_{i}\left(u\right)={e}^{\left[-0.5{\left({d}_{i}\left(u\right)/h\right)}^{2}\right]}$$

${w}_{i}\left(u\right)$ is the geographical weight of the *i*th observation (centroid of the provinces) relative to the location u, ${d}_{i}\left(u\right)$ indicates the Euclidean distance between the *i*th observation and the location u, and h is a parameter named the bandwidth^39^. Although GWR is a significant enhancement compared to SLM and SEM regression models, the scales of relationships between the dependent and explanatory variables are assumed to be constant in GWR. Hence, MGWR is shown as follows^33,40^:

6 $${y}_{i}={\sum }_{j=0}^{m}{\beta }_{bwj}{X}_{ij}+{\varepsilon }_{i},i=\mathrm{1,2},\dots ,n$$

${\beta }_{bwj}$ is the bandwidth used for calibration of the *j*th relationship. Practically, MGWR is a model that could be calibrated using backfitting algorithms. The MGWR can be reformulated as explained in the following equaton^40^.

7 $${y}_{i}={\sum }_{j=0}^{m}{f}_{ij}+\varepsilon $$

${\beta }_{bwj}{X}_{ij}$ is replaced by ${f}_{ij}$, while indicates the *j*th additive term and is a function utilized to *j*th dependent variable of *i*th province. Consequently, the MGWR model incorporates different bandwidths for independent variables. These varying bandwidths capture differences in spatial scales, allowing the model to capture spatial heterogeneity by considering the influence of scale on spatial processes. By incorporating different bandwidths, the MGWR model can effectively account for variations in spatial relationships across different scales, providing a more comprehensive understanding of the spatial processes^33,40^.

In this study, we conducted several statistical tests to assess the validity and robustness of our regression models. Firstly, we employed the Jarque–Bera test to examine the normality of the residuals obtained from the OLS regression^41^. The null hypothesis (p value < 0.05) assumes that the residuals follow a normal distribution, while the alternative hypothesis (p value > 0.05) suggests departures from normality. Secondly, we employed Moran's I test to assess the presence of spatial patterns in the distribution of FMD incidence^42^. Moran's I test helps us determine if there is spatial autocorrelation in the occurrence of FMD incidence, indicating whether neighboring areas exhibit similar levels of FMD incidence. Lastly, we employed the condition number (CN) to measure multicollinearity in the GWR and MGWR regression models^43^. The condition number quantifies the degree of multicollinearity, indicating the potential presence of highly correlated independent variables. By assessing the condition number, we can identify and address issues related to multicollinearity, which can affect the stability and interpretability of regression models.

## Results

Initially, 46 candidate independent variables were subjected to Pearson correlation analysis, which identified four variables with correlations greater than |0.3|. Specifically, these variables were the percentage of the population, precipitation in January, and the percentages of sheep and goats (see Supplemental Fig. 1). Subsequently, the ordinary least squares (OLS) model was applied to these four selected variables to assess their variance inflation factors (VIFs). As shown in Table 2, all four variables exhibited VIF values below 5, indicating no significant multicollinearity issues within the OLS model. Finally, based on these findings, the four selected variables were deemed suitable for inclusion in both the global and local spatial models, which will be further examined in the subsequent analysis.

**Table 2:** Variance inflation factor (VIF) for the final independent variables.

Variable	VIFs
The percentage of population	1.14318
The percentage of sheep	1.17634
The percentage of goats	1.25663
Precipitation in January	1.17050

The OLS model yielded an AIC value of 208.396. Furthermore, the Jarque–Bera statistic (value = 5.804, p = 0.0548) indicated insufficient evidence to conclude that the OLS regression residuals deviate from a normal distribution. Based on Supplemental Table 2, Moran’s I test (I = 0.00078, z = 0.51011, p = 0.60997) suggests that the pattern of FMD incidence does not appear to be significantly different from random. It indicates that there is no compelling evidence of spatial clustering or dispersion in the distribution of FMD incidence.

The *p values* associated with the selected independent variables in Supplemental Tables 3 and 4 for the global models (OLS and SLM) indicate that the percentage of sheep and goats have p values below 0.05. It suggests that these two variables are statistically significant among the independent variables and positively associated with FMD incidence per province.

Based on the results presented in Table 3, the OLS model, with the lowest adjusted ${R}^{2}$ value of 0.43, performed the poorest among the global and local models. However, when accounting for spatial dependence, the SEM and SLM models demonstrated improved performance compared to OLS, with adjusted ${R}^{2}$ values of 0.52 and 0.51, respectively. Moving to the analysis of local spatial differences, GWR and MGWR were utilized. Notably, the local models exhibited substantially improved adjusted ${R}^{2}$ compared to the global models. Specifically, the adjusted ${R}^{2}$ value for GWR was 0.70, indicating a considerable increase in explanatory power. However, MGWR outperformed all other models, achieving the highest adjusted ${R}^{2}$ value of 0.90 and the lowest AIC value of 27.499. These results indicate that MGWR is the most effective model employed in this study, explaining 90% of the total variations in FMD incidence. Additionally, Moran's I test was conducted on the residuals of both GWR (I = − 0.019827, z = 0.188240, p = 0.850688) and MGWR (I = − 0.024190, z = 0.129640, p = 0.896851) indicated statistical insignificance, suggesting a lack of residual spatial autocorrelation in line with the model assumption.

**Table 3:** The value of adjusted ${R}^{2}$ and AIC for global and local approaches in the modeling of FMD in Iran from March 21, 2017, to March 21, 2018.

Parameter/Model	OLS	SLM	SEM	GWR	MGWR
Adjusted ${R}^{2}$	0.43	0.52	0.51	0.70	0.90
AIC	208.396	209.681	208.296	57.795	27.499

The results of the MGWR model are presented in Supplemental Table 5. The optimal bandwidth for the independent variables ranges from 169.740 to 3618.130, indicating that these variables exhibit variation on different spatial scales. Specifically, the percentage of goats and the percentage of sheep are observed at a smaller spatial scale compared to the percentage of the population and the precipitation in January. On the other hand, the GWR model employs a fixed bandwidth of 485.560 for all variables, which does not account for the varying spatial scales of the predictors. Analyzing the spatial variability of local variables value reveals that these predictors primarily have a local impact rather than a global one, further highlighting the localized nature of their influence on FMD incidence.

**Figure 4:**
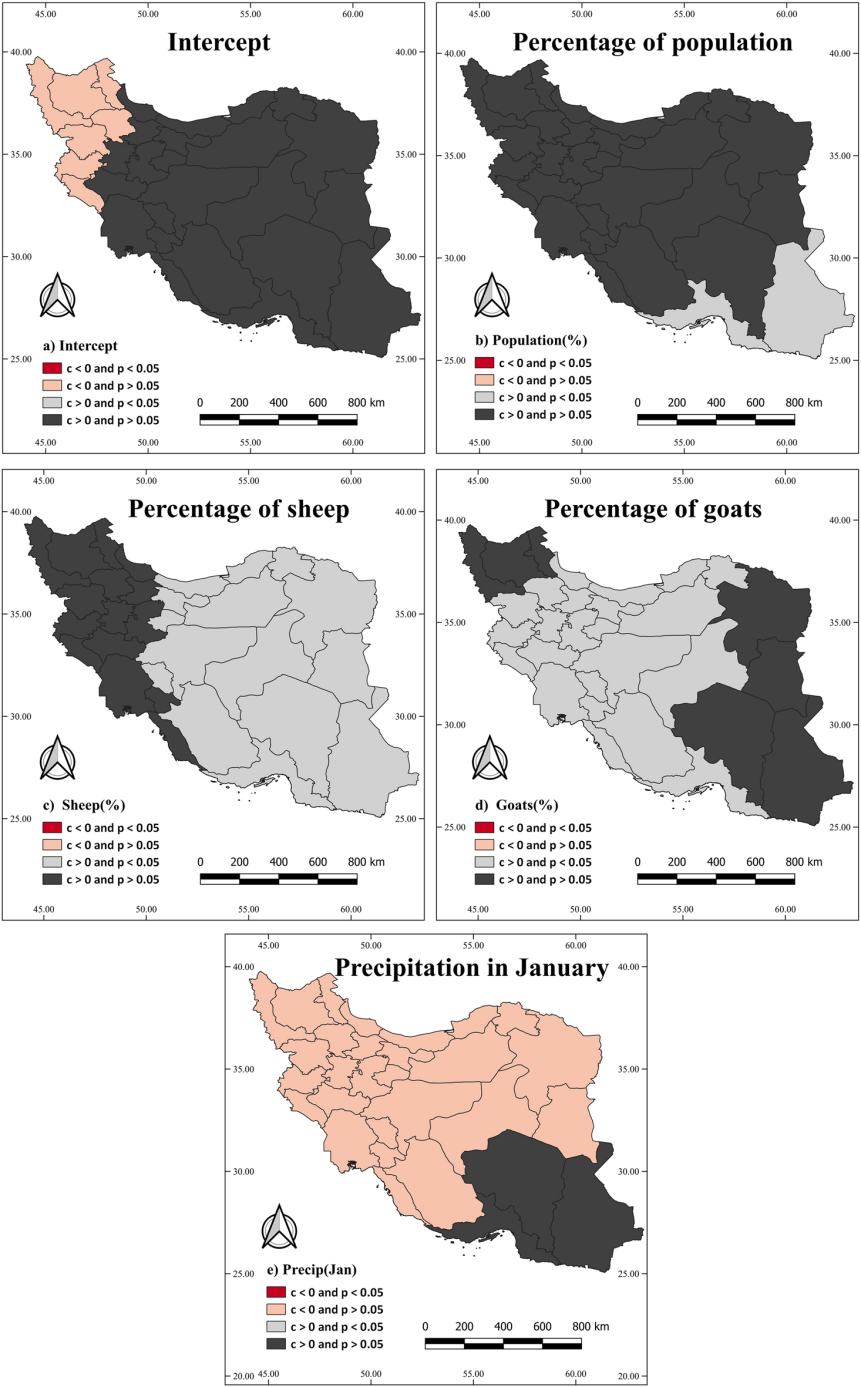
Illustrating the percentage of sheep, goats, population, and precipitation in January associations with the FMD using the GWR model at the provincial level. The “c” and “p” labels indicate the coefficient and the p value*,* respectively.

**Figure 5:**
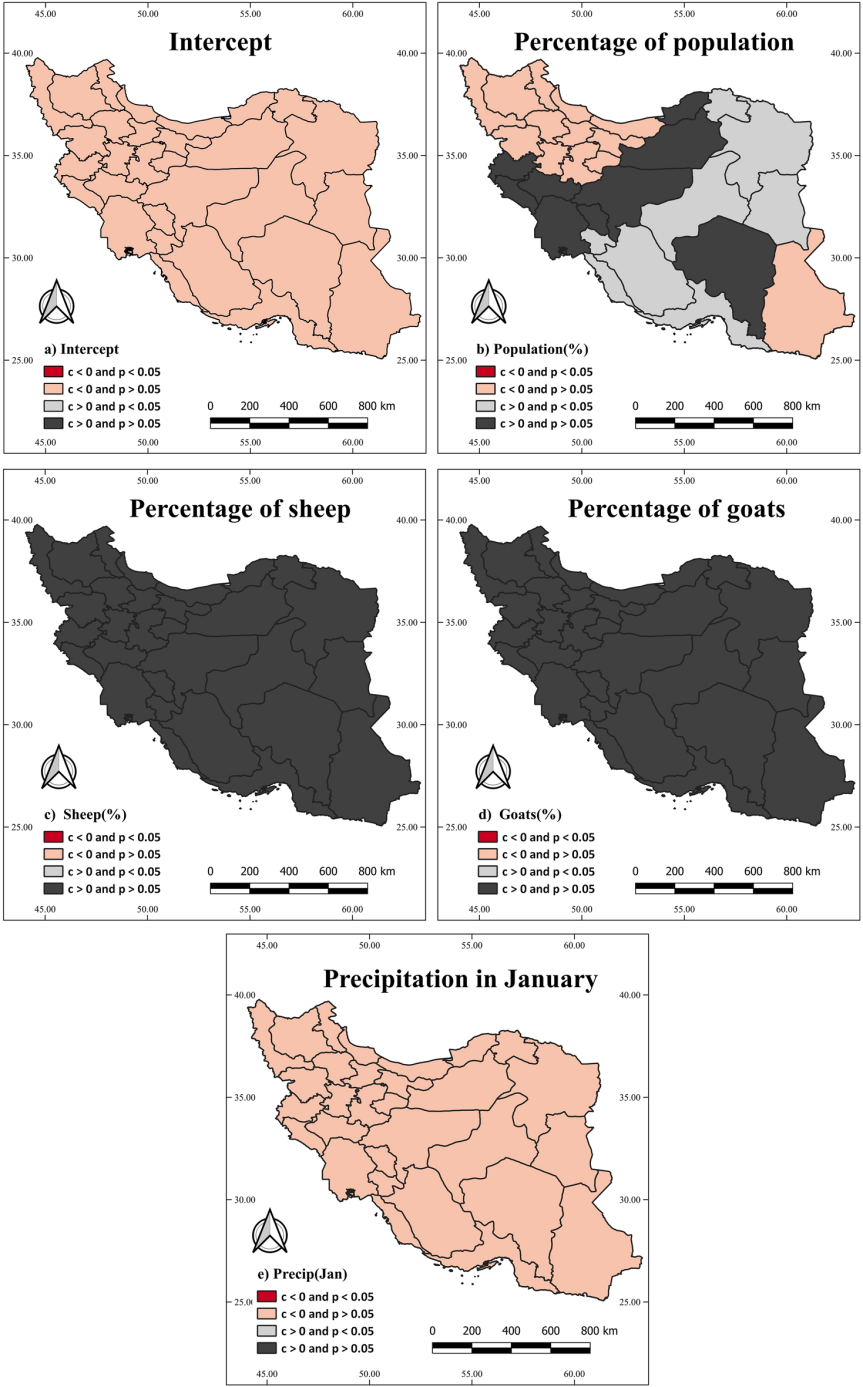
Illustrating the percentage of sheep, goats, population, and precipitation in January associations with the FMD using the MGWR model at the provincial level. The “c” and “p” labels indicate the coefficient and the p value, respectively.

Figures 4 and 5 depict the spatial variation of variables based on the GWR and MGWR models, respectively. In these figures,* p values* less than 0.05 in the provinces indicate statistical significance, and positive coefficients indicate a positive association between a variable and FMD incidence. Specifically, Fig. 4a represents the intercept for the GWR model, while Fig. 5a represents the intercept for the MGWR model. Figure 4b highlights a statistically significant and positive association between the percentage of the population and FMD incidence in southeastern provinces, including Sistan and Baluchestan, as well as Hormozgan provinces. It suggests that a higher population percentage in these regions is associated with an increased risk of FMD.

Figure 4c illustrates a statistically significant and positive association between the percentage of sheep and FMD incidence throughout the country, excluding the western provinces. It suggests that a higher percentage of sheep in most regions of the country is associated with an increased risk of FMD. Figure 4d displays a statistically significant and positive association between the percentage of goats and FMD incidence throughout the country, except in the eastern and northwestern provinces. It indicates that a higher percentage of goats in most regions of the country is associated with an increased risk of FMD. On the other hand, Fig. 4e reveals a statistically insignificant association between precipitation in January and FMD incidence. It suggests that precipitation in January does not significantly contribute to the variation in FMD incidence across the country. Moving on to Fig. 5b demonstrates a statistically significant and positive association between the percentage of the population and FMD incidence in specific provinces, including Busher, Hormozgan, Fars, Yazd, North Khorasan, South Khorasan, and Razavi Khorasan. However, the percentage of the population is not a statistically significant variable in other provinces. Lastly, Fig. 5c–e indicate that the percentage of sheep, goats, and precipitation in January are not statistically significant variables in Iran, according to the MGWR model.

**Figure 6:**
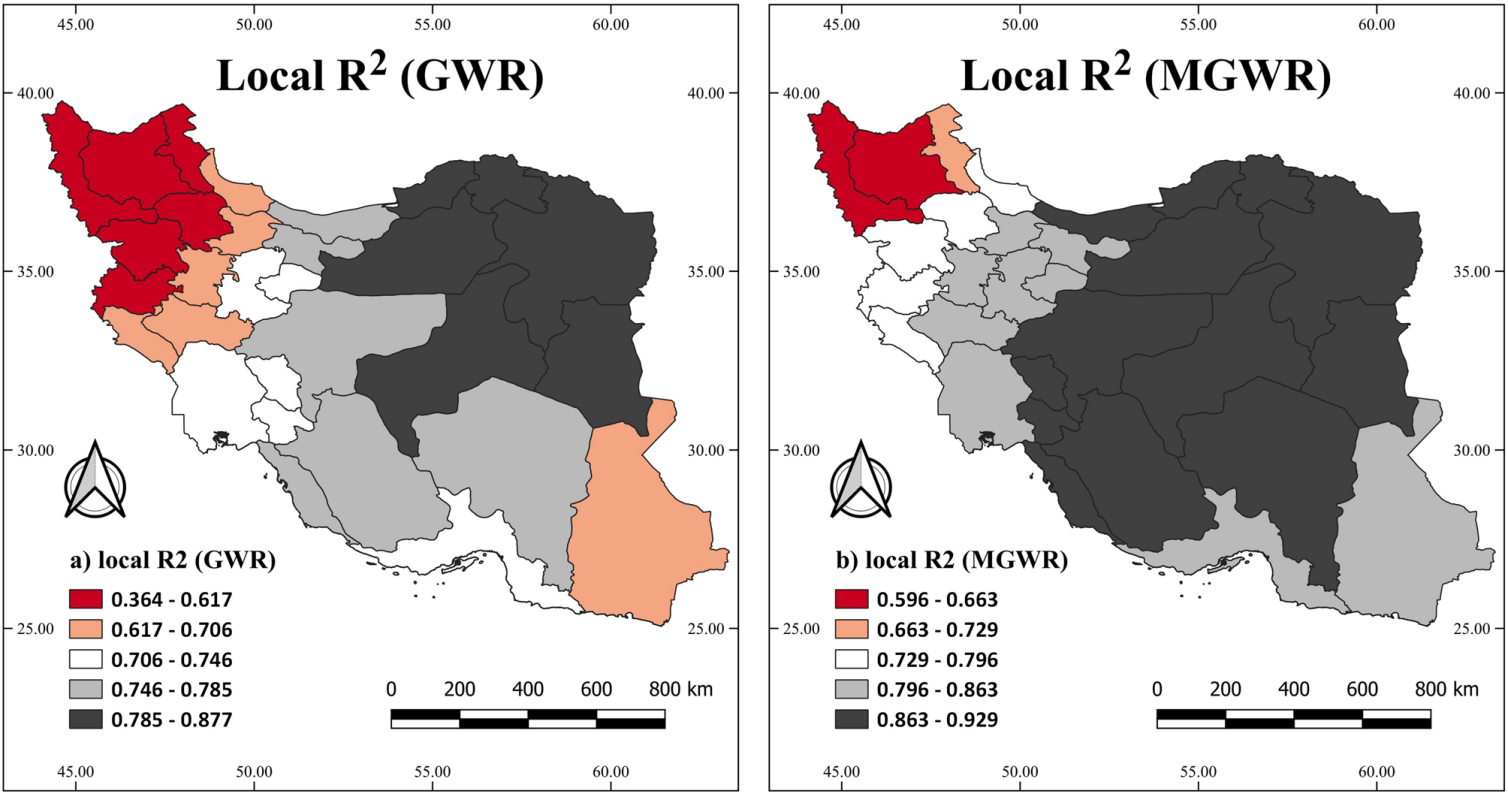
The geographic distribution of the local ${R}^{2}$ of the GWR and MGWR: the association of FMD with the percentage of sheep and goats, population, and the precipitation in January.

Figure 6 shows the local ${R}^{2}$ of GWR and MGWR models used in this study. In both models, several provinces in the northeastern parts had high local ${R}^{2}$, which means the model performs better in these areas. In the northwest, like West Azerbaijan and East Azerbaijan provinces, the value of local ${R}^{2}$ indicates the inadequate performance of the model.

Supplemental Fig. 3 compares the condition numbers (CN) in the GWR and MGWR models. The condition number reflects the degree of collinearity among the explanatory variables in the model. In the GWR model, some provinces in eastern Iran exhibited high condition numbers (CN > 3.47), indicating a high degree of collinearity among the explanatory variables in those provinces. However, the MGWR model showed lower condition numbers than the GWR model, suggesting reduced collinearity among the variables in the MGWR model. It indicates that the MGWR model provides a more reliable and stable estimation of the relationships between the variables in those provinces.

## Discussion

In this study, we employed spatial modeling methods to identify the key factors contributing to FMD incidence at the province level in Iran. Out of the 46 potential variables considered, we selected four variables representing different thematic categories, including environmental, socioeconomic, demographic, and topographic factors. These variables include the percentage of the population, precipitation in January, percentage of sheep, and percentage of goats. We aimed to capture the essential determinants of FMD incidence in Iran by focusing on these variables. Our approach aligns with recent studies that have utilized spatial analysis techniques to investigate FMD patterns and drivers^14,44,45^. Dion and Lambin^46^ conducted a study examining the transmission risk scenarios of FMD in southern Africa, considering climatic, social, and landscape changes, which aligns with our research. Furthermore, another study investigated the impacts of climate change, specifically abrupt temperature changes, on the risk of FMD disease in elephants across Asia and Africa, showcasing the relevance of climate-related factors in understanding FMD dynamics^7^.

In this study, we employed a comprehensive modeling approach that included global aspatial modeling (OLS), global spatial modeling (SEM and SLM), and local spatial modeling (GWR and MGWR) to analyze the spatial distribution of FMD in Iran. Our findings highlight the superiority of the MGWR model over the traditional GWR model in achieving a more precise model fit. By utilizing unique bandwidths for each covariate, MGWR can capture intricate relationships that may be overlooked by the GWR model. Although this approach increases computational complexity, it provides a more nuanced understanding of spatial patterns and influences of each covariate on FMD incidence. In line with our study, previous research by Ye et al.^47^ demonstrated a relationship between urban landscape patterns and infectious disease risk using GWR and OLS modeling techniques. Their findings indicated that GWR outperformed OLS in capturing the spatial variations of disease risk^47^. Zhang et al.^48^ also investigated the spatial distribution of tuberculosis and its association with meteorological factors in mainland China. Their study revealed that GWR was a more suitable modeling approach versus OLS, as indicated by higher adjusted ${R}^{2}$ values and lower AICc scores. The results from Ye et al. and Zhang et al. are consistent with our findings, supporting the notion that local spatial regression methods such as GWR and MGWR yield higher adjusted ${R}^{2}$ values when compared to global regression methods (the OLS, SLM, and SEM)^47,48^.

Following the spatial modeling analysis, our study identified two key variables, the percentage of sheep and goats, which demonstrated a significant impact on disease incidence across most provinces in Iran. Continuous monitoring of these variables is crucial for understanding the dynamics of FMD spread at the provincial level in Iran. These findings align with the results of Begovovea et al.^49^ study, which identified the population of sheep, plus goats, as significant factors influencing FMD prevalence in northern Nigeria (Bauchi, Kaduna, and Plateau states). In contrast, while previous studies have highlighted the significance of climate factors such as precipitation in FMD occurrence, our study did not find precipitation to be a significant variable. This contrasts with the findings of Rahman et al.^50^, who investigated FMD space–time clusters and risk factors in Bangladeshi cattle and buffalo. They highlighted the substantial role of climate, particularly precipitation, in FMD incidence^50^. Jiang et al.^7^ also emphasized the influence of climate change on FMD risk in elephants, noting the importance of precipitation and temperature in this context. Additionally, Lee et al.^51^ studied the temporal patterns and space–time cluster analysis of FMD cases in Vietnam from 2007 to 2017. They identified a higher occurrence of FMD cases during the dry season, from November to March^51^. These variations in findings regarding the significance of precipitation in FMD incidence highlight the importance of considering regional and local factors in disease dynamics. Further research is needed to explore the specific contextual factors that influence FMD transmission patterns in different regions, considering the interplay between climatic variables, host characteristics, and local epidemiological conditions.

### Limitations

There were several limitations to this study. Firstly, the finest spatial granularity for demographic data in Iran was available at the province level due to limited data access. However, obtaining data at the county, district, and farm levels could have provided more accurate and detailed results. Additionally, the unavailability of FMD disease data disaggregated by species restricted the analysis to the provincial level without considering the specific impact of FMD on different livestock species. Access to species-specific data would have facilitated a more comprehensive understanding of the disease dynamics and allowed for targeted prevention and control measures. Secondly, the study was limited regarding the variables used in the modeling process. While efforts were made to include relevant socio-environmental determinants, additional variables such as vaccination-related factors, as observed in previous studies, could have enhanced the analysis. For instance, considering vaccination coverage, particularly the vaccination of calves under 12 months, as an explanatory variable would have provided valuable insights into the effectiveness of vaccination strategies in controlling FMD. Thirdly, the temporal scope of the study was limited to the period from March 21, 2017, to March 21, 2018. A more extended or recent time frame would have allowed for a more comprehensive assessment of FMD incidence trends and patterns in Iran. Incorporating longer-term data could have provided insights into temporal variations and the potential impact of evolving factors on FMD incidence. Lastly, it is essential to acknowledge that the results of this study are generalizable only at the province level. Attempting to conclude sub-province or individual levels may lead to inaccurate inferences due to the potential for ecological fallacy. Therefore, caution should be exercised when extrapolating findings beyond the analyzed spatial scale. Addressing these limitations in future studies, including obtaining data at finer spatial and temporal resolutions, incorporating species-specific FMD information, and expanding the range of variables considered, would further enhance our understanding of FMD dynamics and support more targeted and effective control strategies.

## Conclusion

In this study, we employed global and local spatial models to investigate the key factors influencing the occurrence of FMD in Iran. Our findings revealed that global models performed relatively poorly compared to local models, highlighting the importance of capturing spatial variation in FMD incidence. The MGWR model demonstrated the highest performance, with an adjusted ${R}^{2}$ of 90% among the local models. It emphasizes the significance of considering localized spatial effects when studying FMD incidence. The results highlighted the percentage of sheep and the percentage of goats as the most significant factors among the four selected socio-environmental determinants in explaining FMD incidence across most of the provinces in Iran. It underscores the importance of considering the livestock population when making vaccination-related decisions.

### Supplementary Information

Supplementary Information.

## Data Availability

The datasets used and/or analyzed during the current study are available from the corresponding author on reasonable request.
