# Supplementary material for: Socioeconomic and environmental determinants of foot and mouth disease incidence: an ecological, cross-sectional study across Iran using spatial modeling
Source: Sci Rep. 2023 Aug 19;13:13526. doi: 10.1038/s41598-023-40865-4 (PMC10439931; doi:10.1038/s41598-023-40865-4)
Supplement: Supplementary file 1 — Supplementary Information. [file 41598_2023_40865_MOESM1_ESM.docx]

## Supplementary Tables

**Supplemental Table 1.** Independent variables prepared for each province

| **Variables** | | **Value** | **Variable Unit** |
| --- | --- | --- | --- |
| **V1** | Percentage of population | | % |
| **V2** | Unemployment rate | | % |
| **V3** | Percentage of migrated persons | | % |
| **V4** | Percentage of educated persons | | % |
| **V5** | Percentage of sheep | | % |
| **V6** | Percentage of cows | | % |
| **V7** | Percentage of goats | | % |
| **V8** | Percentage of GDP | | % |
| **V9** | Elevation | | Meters |
| **V10** | Mean NDVI from March 2017 to March 2018 | | Grayscale (0-255) |
| **V11** | Precipitation in January 2018 | | Millimeters |
| **V12** | Precipitation in February 2018 | | Millimeters |
| **V13** | Precipitation in March 2017 | | Millimeters |
| **V14** | Precipitation in April 2017 | | Millimeters |
| **V15** | Precipitation in May 2017 | | Millimeters |
| **V16** | Precipitation in June 2017 | | Millimeters |
| **V17** | Precipitation in July 2017 | | Millimeters |
| **V18** | Precipitation in August 2017 | | Millimeters |
| **V19** | Precipitation in September 2017 | | Millimeters |
| **V20** | Precipitation in October 2017 | | Millimeters |
| **V21** | Precipitation in November 2017 | | Millimeters |
| **V22** | Precipitation in December 2017 | | Millimeters |
| **V23** | Maximum temperature in January 2018 | | ° C |
| **V24** | Minimum temperature in January 2018 | | ° C |
| **V25** | Maximum temperature in February 2018 | | ° C |
| **V26** | Minimum temperature in February 2018 | | ° C |
| **V27** | Maximum temperature in March 2017 | | ° C |
| **V28** | Minimum temperature in March 2017 | | ° C |
| **V29** | Maximum temperature in April 2017 | | ° C |
| **V30** | Minimum temperature in April 2017 | | ° C |
| **V31** | Maximum temperature in May 2017 | | ° C |
| **V32** | Maximum temperature in May 2017 | | ° C |
| **V33** | Maximum temperature in June 2017 | | ° C |
| **V34** | Minimum temperature in June 2017 | | ° C |
| **V35** | Maximum temperature in July 2017 | | ° C |
| **V36** | Minimum temperature in July 2017 | | ° C |
| **V37** | Maximum temperature in August 2017 | | ° C |
| **V38** | Minimum temperature in August 2017 | | ° C |
| **V39** | Maximum temperature in September 2017 | | ° C |
| **V40** | Minimum temperature in September 2017 | | ° C |
| **V41** | Maximum temperature in October 2017 | | ° C |
| **V42** | Minimum temperature in October 2017 | | ° C |
| **V43** | Maximum temperature in November 2017 | | ° C |
| **V44** | Minimum temperature in November 2017 | | ° C |
| **V45** | Maximum temperature in December 2017 | | ° C |
| **V46** | Minimum temperature in December 2017 | | ° C |

**Supplemental Table 2.** Spatial autocorrelation report of FMD incidence.

| Moran's Index: | 0.000788 |
| --- | --- |
| Expected Index: | -0.034483 |
| Variance: | 0.004781 |
| z-score: | 0.510115 |
| p-value: | 0.609971 |

**Supplemental Table 3.** Coefficients and p-values of the OLS model for dependent variables. The variables with (*) are the most significant factors among dependent variables. The parameter $b_{0}$ is explained in equation (1).

| **Variable** | **Coefficient** | **P-value** |
| --- | --- | --- |
| $b_{0}$ | -2.98655 | 0.45210 |
| The percentage of sheep * | 1.58503 | 0.01001 |
| The percentage of goats * | 0.844726 | 0.04510 |
| The percentage of population | 0.438214 | 0.32792 |
| Precipitation in January | -0.0811962 | 0.42434 |

**Supplemental Table 4.** Coefficients and p-values of the SLM model for dependent variables. The variables with (*) are the most significant factors among dependent variables. The parameter $b_{0}$ and $\rho$ are explained in equation (1).

| **Variable** | **Coefficient** | **P-value** |
| --- | --- | --- |
| $b_{0}$ | 0.0287 | 0.99480 |
| The percentage of sheep * | 1.49632 | 0.00365 |
| The percentage of goats * | 0.790592 | 0.02788 |
| The percentage of population | 0.360996 | 0.37318 |
| Precipitation in January | -0.126036 | 0.18958 |
| $\rho$ | -0.232249 | 0.35185 |

**Supplemental table 5.** Estimation results of the MGWR

| Variables | Mean | St.Dev | Min | Max | Bandwidth |
| --- | --- | --- | --- | --- | --- |
| Intercept | -0.025 | 0.001 | -0.026 | -0.024 | 3617.950 |
| The percentage of sheep | 0.316 | 0.265 | -0.006 | 0.860 | 389.320 |
| The percentage of goats | 0.335 | 0.440 | -0.136 | 1.897 | 169.740 |
| The percentage of population | 0.021 | 0.000 | 0.021 | 0.021 | 3618.040 |
| Precipitation in January | -0.178 | 0.001 | -0.179 | -0.177 | 3618.130 |

## Supplementary Figures


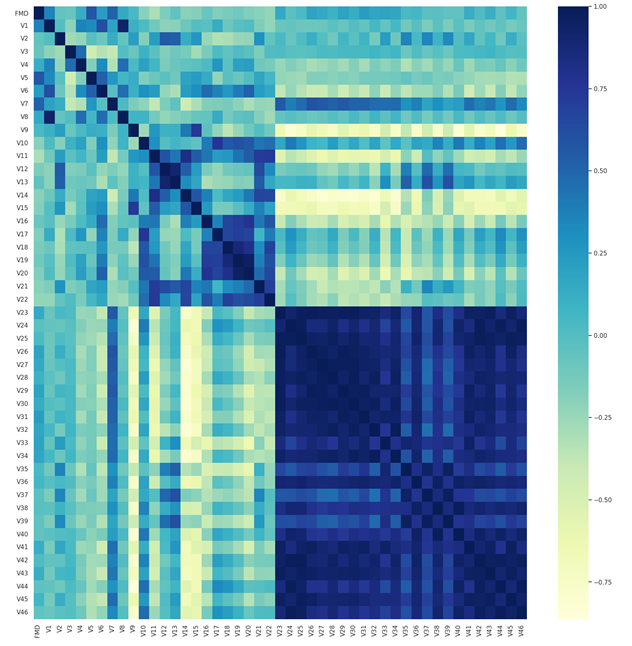
**Supplemental Figure 1**. Pearson correlation matrix. The variables which had correlation more than |0.3| for dependent variable were selected in the first step. See supplemental Table 1 to find the name of variables (from V1 to V46).


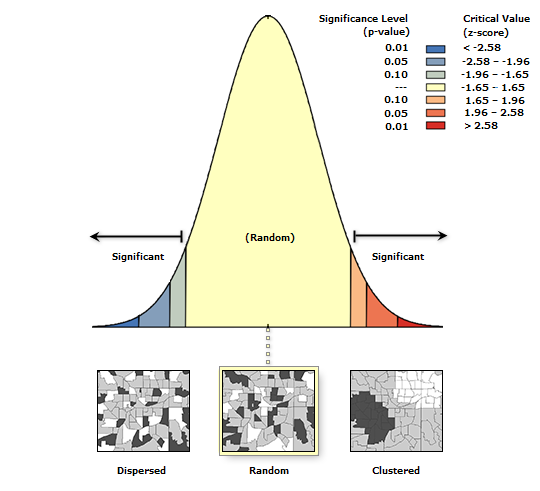


**Supplemental Figure 2**. Given the z-score of 0.5101, the pattern of FMD incidence in Iran does not appear to be significantly different than random.


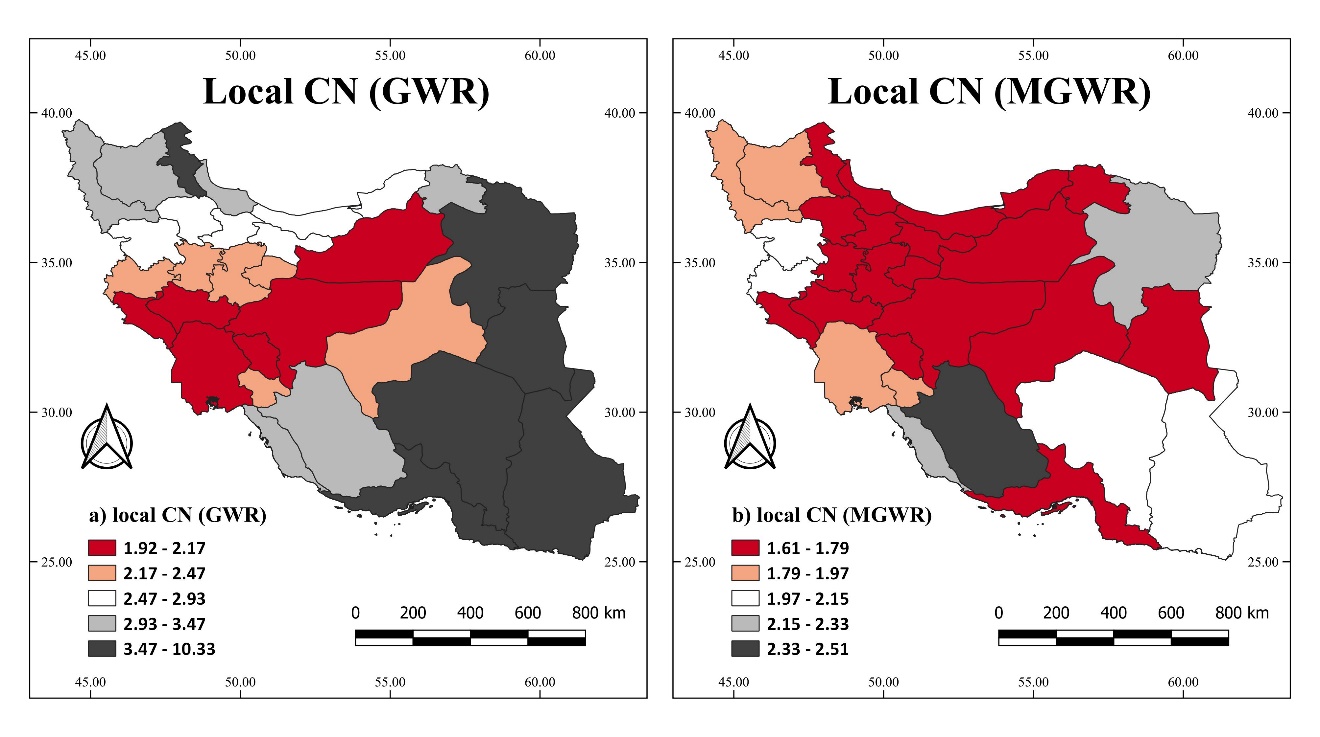


**Supplemental Figure 3**. Diagnostic tests of the local collinearity for the GWR (left) and the MGWR (right)
